# Cardiac remodeling and arrhythmia in a mouse model of *Depdc5* haploinsufficiency

**DOI:** 10.1002/epi.70244

**Published:** 2026-04-09

**Authors:** Roberto Ramos‐Mondragon, Shuyun Wang, Qinghua Liu, Chunling Chen, Alexander M. Greiner, Abigail M. Marx, Maya Shih, Jack M. Parent, Barry London, Lori L. Isom

**Affiliations:** ^1^ Department of Pharmacology University of Michigan Medical School Ann Arbor Michigan USA; ^2^ Department of Internal Medicine–Division of Cardiovascular Medicine University of Iowa Carver College of Medicine Iowa City Iowa USA; ^3^ Department of Neurology University of Michigan Medical School Ann Arbor Michigan USA; ^4^ Michigan Neuroscience Institute University of Michigan Medical School Ann Arbor Michigan USA; ^5^ VA Ann Arbor Healthcare System Ann Arbor Michigan USA

**Keywords:** cardiac arrhythmia, developmental and epileptic encephalopathy, mouse model

## Abstract

**Objective:**

Some ion channel genes linked to developmental and epileptic encephalopathy (DEE) are also linked to cardiac arrhythmia, leading to the hypothesis that predisposition to cardiac arrhythmias may contribute to the complex disease presentation of DEE and possibly to the mechanism of sudden unexpected death in epilepsy. However, channelopathies represent only ~25% of the genetic epilepsies. The remainder arise from variants in non‐ion‐channel genes, moving the disorder beyond channelopathies. Despite evidence that some non‐ion‐channel variants are linked to sudden cardiac death, we have little information on whether non‐ion‐channel DEE variants can also result in cardiac phenotypes.

**Methods:**

Here, we investigated the DEE gene, *DEPDC5,* which is expressed in both brain and heart. We studied the cardiac phenotype of a *Depdc5* haploinsufficient mouse model that genetically mimics *DEPDC5* patients.

**Results:**

*Depdc5* ^*+/−*^ mice showed increased susceptibility to ventricular arrhythmias, systolic dysfunction, and ventricular fibrosis, as well as shortened action potential duration, increased levels of sodium and potassium channel proteins, and increased sodium current and transient outward potassium current densities in acutely isolated ventricular myocytes. Thus, at least in mice, *Depdc5* variants impact heart as well as brain excitability.

**Significance:**

These data strengthen the hypothesis that cardiac arrhythmias may contribute to the complex presentation of DEE and expand our previous work showing cardiac arrhythmia in multiple mouse and human channelopathy models to include a model of a non‐ion‐channel variant. Although this work may have implications for *DEPDC5* patients, critical differences between mouse and human cardiac physiology complicate the translation of mouse data to human disease. Essential next steps must include investigation of *Depdc5* function in higher vertebrate models that more accurately mimic human physiology, as well as longitudinal patient natural history studies that monitor cardiovascular health, to test the hypothesis that DEE variants resulting in *DEPDC5* haploinsufficiency may predispose patients to cardiac arrhythmias.

**Key points**

- We investigated a gene linked to developmental and epileptic encephalopathy, *DEPDC5*, which is expressed in both brain and heart.
- We studied the cardiac phenotype of a *Depdc5* haploinsufficient mouse model that genetically mimics *DEPDC5* patients.
- *Depdc5* ^*+/−*^ mice showed increased susceptibility to ventricular arrhythmias, systolic dysfunction, and ventricular fibrosis.
- Mouse ventricular myocytes showed alterations in action potentials, expression of ion channel proteins, and ionic current densities.
- Thus, at least in mice, *Depdc5* variants impact heart as well as brain excitability.

## 1 INTRODUCTION

A subset of genes linked to developmental and epileptic encephalopathy (DEE) are also linked to cardiac arrhythmia, reflecting their coexpression in brain and heart. This knowledge, especially for channelopathy genes, has led to the hypothesis that predisposition to cardiac arrhythmias may contribute to the complex disease presentation of DEE and possibly to the mechanism of sudden unexpected death in epilepsy (SUDEP).^1^, ^2^, ^3^, ^4^, ^5^, ^6^ Interestingly, when SUDEP is compared to sudden cardiac death (SCD) in long QT syndrome (LQT), especially in LQT type 3 linked to variants in the voltage‐gated sodium channel (VGSC) gene *SCN5A*, there are parallels in the circumstances of death.^7^

Our previous work focused on channelopathies like Dravet syndrome (DS),^8^ a DEE with SUDEP risk of up to 20%.^1^ More than 90% of DS is caused by variants in *SCN1A*, encoding the VGSC Na_v_1.1 α subunit.^9^ A smaller cohort have biallelic variants in *SCN1B*, encoding the VGSC β1 subunits.^10^, ^11^, ^12^ A related DEE is linked to variants in *SCN8A*, encoding Na_v_1.6.^13^ Importantly, *SCN1A*, *SCN8A*, and *SCN1B* are expressed in brain and heart of humans and mice.^14^, ^15^, ^16^ We demonstrated cardiac arrhythmias due to intrinsic differences in cardiac myocyte (CM) excitability in these mouse models of channelopathy‐linked DEEs as well as substrates for arrhythmia in DS patient‐derived induced pluripotent stem cell CMs.^14^, ^15^, ^17^, ^18^, ^19^

Unbiased exome sequencing has revealed the breadth of biological processes implicated in epilepsy, showing that channelopathies represent only ~25% of the genetic epilepsies.^20^ The remainder arise from variants in more than 100 non‐ion‐channel genes, highlighting the diversity of epilepsy mechanisms and moving the disorder beyond channelopathies. Despite this rapid progress and evidence that non‐ion‐channel gene variants are also linked to SCD,^21^, ^22^, ^23^ we have little information on whether non‐ion‐channel DEE gene variants also result in cardiac phenotypes.

Pathogenic variants in *DEPDC5*, encoding DEP domain‐containing protein 5, are associated with a spectrum of epilepsies.^24^ *DEPDC5* pathogenic variants are monoallelic loss of function (LOF) with a possible second hit causing disease.^25^, ^26^, ^27^, ^28^, ^29^ Ubiquitously expressed throughout the body,^30^ DEPDC5 is a guanosine triphosphatase (GTPase) member of the GATOR1 complex, an inhibitory regulator of the mammalian target of rapamycin (mTOR) pathway that is critical for cell growth and metabolism in all eukaryotic cells. The GATOR1 complex regulates cellular metabolism in response to nutrient stress.^27^ When essential amino acids are low, the GATOR1 complex prevents mTOR complex 1 (mTORC1) activation, resulting in inhibition of the mTOR pathway.^31^, ^32^, ^33^ Thus, LOF *DEPDC5* variants result in mTOR pathway hyperactivation. Dysregulation of mTORC1 has been linked to epilepsy, neurodegeneration, cancer, diabetes, cardiac hypertrophy, and heart failure.^24^, ^34^, ^35^

Here, we investigated the cardiac phenotype of a floxed *Depdc5* mouse model crossed with EIIa‐Cre to generate ubiquitous *Depdc5* haploinsufficiency to genetically mimic *DEPDC5* patients. We show that *Depdc5* haploinsufficiency in mice results in cardiac dysfunction in the complete absence of seizures and SUDEP, implying that *DEPDC5* patients may develop underlying cardiac disease in addition to seizures, and that, although more studies are needed, the combination of seizures and cardiac arrhythmia may synergize to result in a rare terminal event.

## 2 MATERIALS AND METHODS

### 2.1 Study approval

All animal procedures were approved by the University of Michigan institutional animal care and use committee (PRO00010562).

### 2.2 Generation of mice

*C57BL/6N‐Depdc5* ^*tm1a(EUCOMM)Hmgu/H*^ (EM:08844, HAR:006793) mice were used previously to generate neuron‐specific conditional knockout mice that showed spontaneous seizures and premature death.^36^, ^37^ For the studies described here, *C57BL/6N‐Depdc5* ^*tm1a(EUCOMM)Hmgu/H*^ mouse sperm were cryorecovered at the Jackson Laboratory into C57BL/6J mice. The splice acceptor‐LacZ construct and Neo selection cassette were deleted by breeding to Rosa26‐FLP mice (JAX:012930) on the C57BL/6J background, which constitutively express Flp recombinase. Flp recombinase was subsequently bred out of the line by backcrossing five times to C57BL/6J mice. The resulting mice were then backcrossed for two additional generations with B6.FVB‐Tg(EIIa‐cre)C5379Lmgd/J mice (JAX:003724) on the C57BL/6J background to delete *Depdc5* ubiquitously and generate *Depdc5* ^*+/Fl Cre*^ mice. In total, the mouse line was backcrossed for seven generations to the C57BL/6J background. To breed mice for study, heterozygous *Depdc5* ^*+/Fl Cre*^ mice were intercrossed. Polymerase chain reaction (PCR) genotyping of the littermate pups resulted in a ~ 950‐base pair (bp) band indicating wild type (*Depdc* ^*+/+*^) or ~ 950‐bp plus ~600‐bp bands indicating heterozygosity (*Depdc5* ^*+/Fl Cre*^). Homozygous pups (*Depdc5* ^*Fl Cre/Fl Cre*^), which would have generated only the 600‐bp PCR band, were not observed in any litters, suggesting embryonic lethality, consistent with previous work.^38^ Heterozygous *Depdc5* ^*+/Fl Cre*^ mice did not exhibit behavioral seizures or SUDEP. *Depdc* ^*+/+*^ and *Depdc5* ^*+/Fl Cre*^ littermates were used in all experiments by investigators blinded to genotype.

PCR primers used for genotyping were as follows: *Depdc5* forward: 5′‐CTT CTC TTA ACT ACT GGC CAT CTC‐3′ and *Depdc5* reverse: 5′‐TCA GGG AAC TCT AAC ATC CTT TTC TGG‐3′.

Animals were housed in the Unit for Laboratory Animal Medicine at the University of Michigan Medical School. Male and female mice at ~8 months of age were used in all experiments.

### 2.3 Electrocardiogram and programmed electrical stimulation

Electrocardiograms (ECGs) were recorded on anesthetized mice as in Mondragon et al.^39^ Anesthesia was induced with 5.0%vol isoflurane and maintained with 2.0%vol isoflurane in a continuous flow of 100% O_2_ at .5 L/min. Mice were placed on a temperature‐regulated operating table. Platinum electrodes were inserted subcutaneously in the limbs and connected to a custom ECG amplifier for standard leads I and II. ECG parameters, analyzed offline, included heart rate, P‐wave duration, R‐R, Q‐R‐S, P‐R, and QTc intervals (analyzed using the Mitchell formula: QTc = QT/√(RR/100)). In a separate group of mice, a 1.1‐Fr Octapolar stimulation‐recording catheter (Scisense, EP catheter) was inserted through the jugular vein and advanced into the right atrium and ventricle. Ventricular electrical stimulation was performed at twice the threshold of capture. Sinus node recovery time was measured by delivering 18 pacing stimuli at fixed cycle lengths of 100 ms (CL_100_) and 80 ms (CL_80_). Ventricular refractory period (VRP) was determined using a standard S1–S2 protocol; 18 S1 stimuli were delivered at CL_100_ and CL_80_, each followed by a single S2 stimulus. S2 intervals were progressively shortened in 2‐ms decrements, from 40 ms to 16 ms. VRP was defined as the longest S2 interval that failed to elicit ventricular capture. Intervals were progressively shortened until failure to capture, at which point the ventricular effective refractory period was determined. This protocol was repeated three times to ensure reproducibility and to evaluate susceptibility to ventricular tachycardia (VT). Ventricular arrhythmias were defined as nonsustained VT, characterized by three or more consecutive ventricular depolarizations with abnormal QRS morphology distinguishable from baseline rhythm, or ventricular fibrillation, defined as rapid, disorganized electrical activity lacking discernible QRS complexes and lasting at least 1 s.

### 2.4 Echocardiography

Cardiac function was evaluated using a Vevo F2 LT imaging system (FUJIFILM VisualSonics) with a 46‐MHz linear transducer. Mice were anesthetized with 1.5% isoflurane and maintained on a heating pad at physiological temperature. Standard parasternal long‐ and short‐axis views were obtained. M‐mode recordings from the short‐axis view at the midpapillary level measured left ventricular internal diameter at diastole (LVIDd) and left ventricular internal diameter at systole (LVIDs). Left ventricular end‐diastolic volume (LVEDv), left ventricular end‐systolic volume (LVESv), stroke volume, cardiac output (CO), ejection fraction (EF%), and fractional shortening (FS%) were calculated using standard formulas assuming a prolate‐ellipsoid left ventricle (LV) geometry. Pulsed‐wave (PW) Doppler imaging was performed at the level of the mitral inflow to assess diastolic function. E and A wave peak velocities were recorded, and E/A ratio was calculated. Isovolumic relaxation time (IVRT) was measured from cessation of the aortic outflow to the onset of mitral inflow. Myocardial performance index (MPI) was calculated using the Tei index: MPI = (IVCT + IVRT) / AET, where IVCT is the isovolumic contraction time and AET is aortic ejection time, obtained from Doppler tracings. All measurements were averaged over three cardiac cycles and analyzed in a blinded fashion. Data were processed using built‐in measurement software.

### 2.5 Picrosirius Red staining

Picrosirius Red staining was performed in cardiac tissue sections to assess collagen deposition.^20^ Images were acquired with Aperio Digital Pathology Slide Scanners (Aperio GT 450 DX, Leica). Quantification of collagen content was performed using ImageJ (National Institutes of Health) on at least six nonoverlapping fields per section, with the collagen‐stained area expressed as a percentage of the total tissue area. Data are presented as mean ± SEM.

### 2.6 Isolation of mouse ventricular cardiomyocytes

Ventricular CMs were acutely isolated using a Langendorff‐free protocol.^40^ Following euthanasia, the descending aorta was transected. Within 1 min, Hank balanced salt solution (HBSS) containing 10 mmol·L^−1^ hydroxyethylpiperazine ethane sulfonic acid (HEPES), 1 mmol·L^−1^ MgCl₂, and .5 mmol·L^−1^ EDTA was flushed into the right ventricle (RV). EDTA‐free HBSS with Collagenase II (Worthington; 280–285 U/mg) was injected into the left ventricle. Once myocytes began to emerge, cardiac chambers were separated, minced into ~1‐mm^3^ fragments with microforceps, and gently triturated. Digestion was halted with 10% fetal bovine serum, and the cell suspension was filtered through a 100‐μm strainer. Calcium was reintroduced stepwise until 1.0 mmol·L^−1^. Only quiescent, rod‐shaped myocytes were selected for electrophysiological experiments.

### 2.7 Whole cell voltage clamp recordings

Whole cell voltage clamp recordings in acutely isolated ventricular CMs were performed as in Ramos‐Mondragon et al.^40^ with an Axopatch 700B amplifier (Molecular Devices) and pClamp (version 11, Axon Instruments) using fire‐polished pipettes with resistance of 1.5–2.5 mΩ. After establishing whole cell configuration, membrane capacitive components were eliminated, and series resistance was compensated. Sodium (*I*_Na_) and L‐type Ca2+ (*I*_Ca,L_) currents were recorded using the P/4 protocol. Currents were normalized to membrane capacitance.

*I*_Na_ was measured using bath solution containing (in mmol·L^−1^) 10 NaCl, 110 CsCl, 2 MgCl_2_, .2 CdCl_2_, 1 CaCl_2_, 10 HEPES, 20 tetraethylammonium chloride (TEA‐Cl), and 10 glucose (pH = 7.35 with CsOH, osmolarity = ~310 mOsm). Internal solution contained (in mmol·L^−1^) 1 NaCl, 10 ethyleneglycoltetraacetic acid (EGTA), 10 HEPES, 1.0 MgCl, 5 adenosine 5′‐triphosphate di(tris) salt hydrate, .02 Na_2_GTP (pH = 7.2 with CsOH, osmolarity = ~285). *I*_Na_ was recorded in response to a series of voltage steps from −120 mV to +30 mV, in 5‐mV or 10‐mV increments, from a holding potential of −120 mV, applied for 200 ms.^40^ A step back to −20 mV for 200 ms was used to assess the voltage dependence of inactivation. Na^+^ conductance at each test voltage was determined using the equation G_Na_ = *I*_Na_/(*V* − E_Na_), where E_Na_ is the sodium current reversal potential. Peak G_Na_ (G_max_) was plotted as a function of voltage to produce activation curves. *I*_Na_ was normalized to the maximum elicited current and plotted against the conditioning voltage to yield inactivation curves. Both curves were fitted to the Boltzmann function: G/G_max_ or *I*/I_max_ = 1/(1 + exp.[(*V* − *V*_½_)/*k*]), where G/G_max_ is the normalized activation and *I*/I_max_ is normalized inactivation, *V*_½_ is the voltage of half‐maximal activation or inactivation, *k* is the slope factor, and *V* is the test voltage.

*I*_Ca,L_ was measured from −50 to 50 mV in steps of 10 mV to generate an *I*–*V* curve. A conditional prepulse to −30 mV was used to inactivate Na^+^ channels. Bath solution contained (in mmol·L^−1^) 137 NaCl, 5.4 CsCl, 1 MgCl_2_, 1.8 CaCl_2_, 10 HEPES, and 2 4‐aminopyridine (pH 7.4 with CsOH); 35 μmol·L^−1^ of tetrodotoxin was added to the bath solution to ensure the elimination of *I*_Na_. Internal solution contained (in mmol·L^−1^) 120 CsCl‐Asp, 10 EGTA‐Cs, 1 MgCl_2_, 1 Mg‐adenosine triphosphate (ATP), 10 TEA‐Cl, and 10 HEPES (pH 7.2 with CsOH). Activation and inactivation curves were fitted to the Boltzmann equation, as described for *I*_Na_ recordings.

Transient outward (*I*_to_) and sustained (*I*_KSUS_) potassium currents were measured using repetitive squared 300‐ms pulses from −40 to 60 mV. Potassium current (*I*_K_) collected in the last 50 ms of the recording was defined as *I*_KSUS_. The difference between *I*_KSUS_ and peak current collected in the first 50 ms of the recordings was defined as *I*_to_. Inward *I*_K_ was recorded from −140 to −30 mV before and after the perfusion of BaCl_2_ (500 μmol·L^−1^). *I*_K1_ was defined as *I*_K_ that was sensitive to barium. Internal solution for *I*_K_ contained (in mmol·L^−1^) 135 KCl, 5 K_2_‐ATP, 10 EGTA‐K, and 10 HEPES (pH = 7.2 with KOH, osmolarity = ~310 mOsm). Bath solution contained (in mmol·L^−1^) 5.3 KCl, 4.1 NaHCO_3_, 138 NaCl, and 100 CaCl_2_ (pH = 7.2 with KOH, osmolarity = ~295 mOsm); 250 μmol·L^−1^ of CdCl_2_ and 10 μmol·L^−1^ of nifedipine were added to the bath solution to block I_Ca_.

### 2.8 Action potential recordings

The threshold for action potential (AP) initiation in acutely isolated ventricular CMs was determined by applying 2‐ms incremental current pulses from 100 to 1500 pA. Steady state AP capture was obtained by applying current pulses at 1.5× the threshold. APs were recorded at 1.0 Hz at room temperature. Bath solution contained (in mmol·L^−1^) 135 NaCl, 4 KCl, 1.8 CaCl2, 1 MgCl2, 10 HEPES, 1.2 NaH_2_PO_4_, 10 glucose (pH 7.35 with NaOH, osmolarity = ~290 mOsm). Internal solution contained (in mmol·L^−1^) 130 K‐aspartate, 10 KCl, 9 NaCl, .33 MgCl2, 5 Mg‐ATP, .1 GTP, 10 HEPES, 10 glucose (pH 7.2 with KOH, osmolarity = ~290 mOsm). Resting membrane potential (RMP) was determined under current clamp at zero current.

### 2.9 RNA isolation and quantitative reverse transcriptase PCR

Total RNA was extracted from whole mouse ventricles using the Qiagen RNeasy Fibrous Tissue Mini Kit. cDNA was synthesized from 1 μg of total RNA using qScript cDNA Synthesis Kit (QuantaBio, 95 047–100). Quantitative (q)PCR was performed with SYBR Green Master Mix (Applied Biosystems) on a QuantStudio 7 Flex Real‐Time PCR System (Applied Biosystems). Relative gene expression was calculated using the ΔΔCt method and normalized to *Gapdh*. All assays were conducted in technical duplicates with *n* = 8 biological replicates per group. Data are presented as fold change in gene expression ± SEM. Significance of comparisons between genotypes was determined using Student *t*‐test for comparisons between two variables, or one‐way analysis of variance with Tukey post hoc comparison test for comparisons involving more than two variables or Fisher exact test to compare two categorical variables.

### 2.10 Western blot analysis

Ventricular tissues were rapidly dissected from *Depdc5* ^*+/+*^ and *Depdc5* ^*+/Fl Cre*^ mice and homogenized in ice‐cold radioimmunoprecipitation assay buffer supplemented with protease and phosphatase inhibitors. Protein concentration was measured using a bicinchoninic acid assay (Thermo Fisher). Equal protein amounts (30 μg) were loaded onto 4%–15% TGX gradient gels (Bio‐Rad) and separated under reducing conditions. Protein transfer was performed using the Bio‐Rad Trans‐Blot Turbo semidry transfer system onto nitrocellulose membranes. Membranes were blocked for 1 h at room temperature in 5% nonfat dry milk in Tris‐buffered saline with Tween, then incubated overnight at 4°C with primary antibodies against Nav1.5 (1:1000, Abcam ab300048) or Kv4.2 (1:1000, K57/1, Antibodies Incorporated 75–016). After washing, membranes were incubated with fluorescence‐conjugated secondary antibodies (1:1000) for 1 h at room temperature and then visualized by using an iBright FL1000 imaging system (Invitrogen). Protein expression levels were quantified using iBright analysis software (Invitrogen) and normalized to the level of glyceraldehyde‐3‐phosphate dehydrogenase (GAPDH; 1:1000, anti‐GAPDH antibody [EPR16891] Abcam ab181602) for each sample.

### 2.11 Statistical analyses

Statistical test information is presented in each figure legend. Probability values of <.05 were considered significant.

## 3 RESULTS

### 3.1 *Depdc5* ^*+/Fl Cre*^ mice have normal heart size and electrocardiogram properties

Results from surface ECG leads I and II are presented in Table S1. We found no genotypic differences in heart rate, p‐wave duration, P‐R internal, QRS, or QT intervals. Body weight (BW), heart weight (HW), and HW/BW ratio were comparable between genotypes (Table S1).

### 3.2 *Depdc5* ^*+/Fl Cre*^ mice have increased susceptibility to ventricular arrhythmia

**FIGURE 1:**
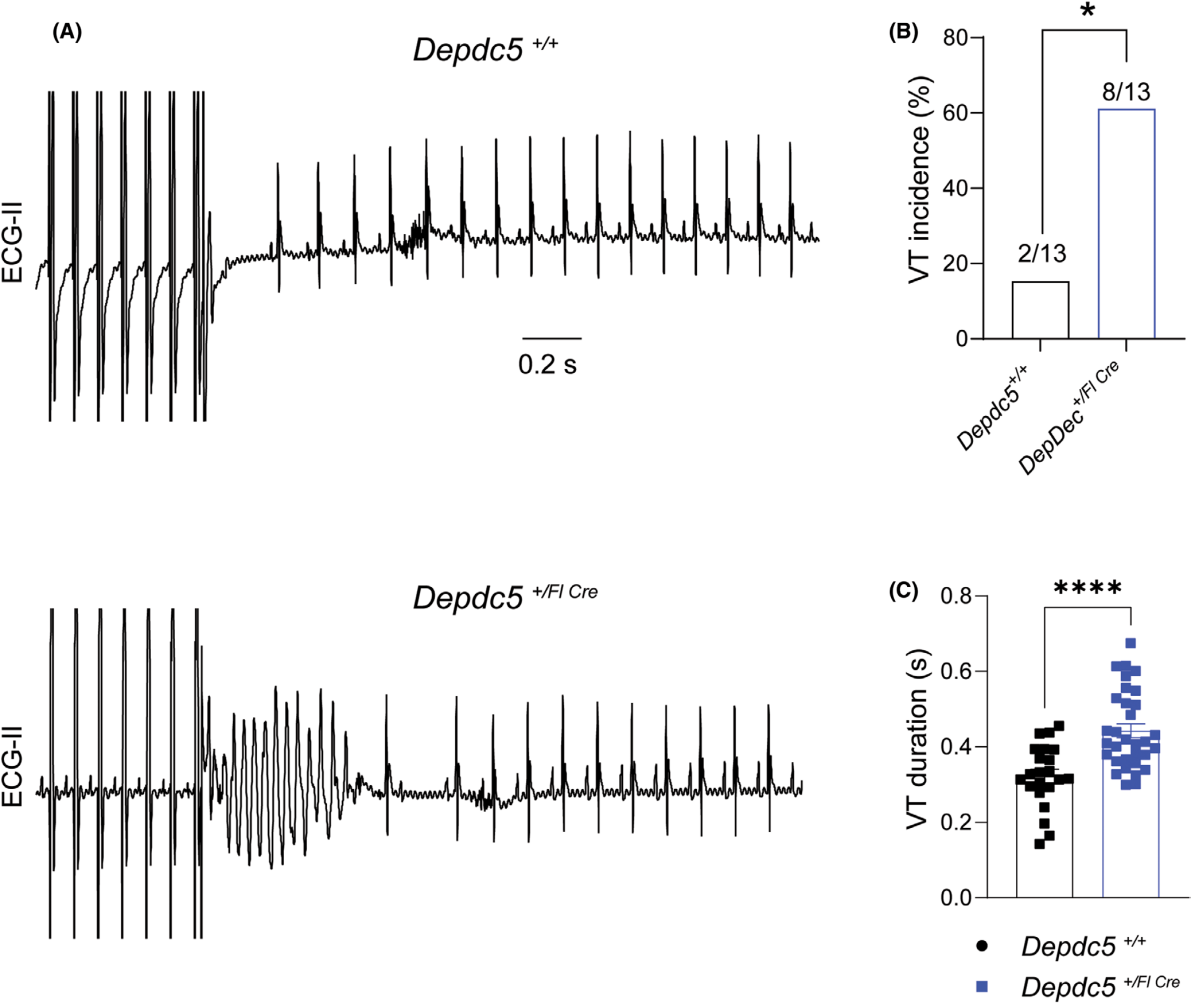
Electrophysiological assessment of cardiac arrhythmias in *Depdc5* ^*+/Fl Cre*^ mice. (A) Representative electrocardiographic (ECG) lead II recordings from postnatal day 230–260 anesthetized *Depdc5* ^*+/+*^ and *Depdc5* ^*+/Fl Cre*^ mice during extra ventricular stimulation. Following electrical stimulation, the *Depdc5* ^*+/Fl Cre*^ mouse developed an episode of ventricular arrhythmia, whereas the *Depdc5* ^*+/+*^ mouse resumed normal sinus rhythm. (B) Ventricular arrhythmia incidence. (C) Duration of ventricular tachycardia (VT) episodes. *Depdc5* ^*+/Fl Cre*^ mice exhibited high incidence and longer VT episodes compared to controls. *p < .05 using Fisher exact test for comparisons of arrhythmia incidence among groups; ****p < .0001 using unpaired two‐tailed Student *t*‐test.

We performed intracardiac recordings to determine whether ubiquitous *Depdc5* haploinsufficiency predisposes mice to arrhythmogenesis. Following programmed electrical simulation, *Depdc5* ^*+/+*^ mice returned to normal sinus rhythm, whereas *Depdc5* ^*+/Fl Cre*^ mice frequently developed ventricular arrhythmia (Figure 1A). Eight of 13 *Depdc5* ^*+/Fl Cre*^ mice developed arrhythmias compared to two of 13 *Depdc5* ^*+/+*^ mice (*p* < .05; Figure 1B). Ventricular arrhythmias in *Depdc5* ^*+/Fl Cre*^ mice were longer in duration than controls (.44 ± .018 s vs. .32 ± .018 s, *p* < .0001; Figure 1C). Thus, *Depdc5* haploinsufficiency increases susceptibility to ventricular arrhythmias.

### 3.3 *Depdc5* ^*+/Fl Cre*^ mice show systolic dysfunction

**FIGURE 2:**
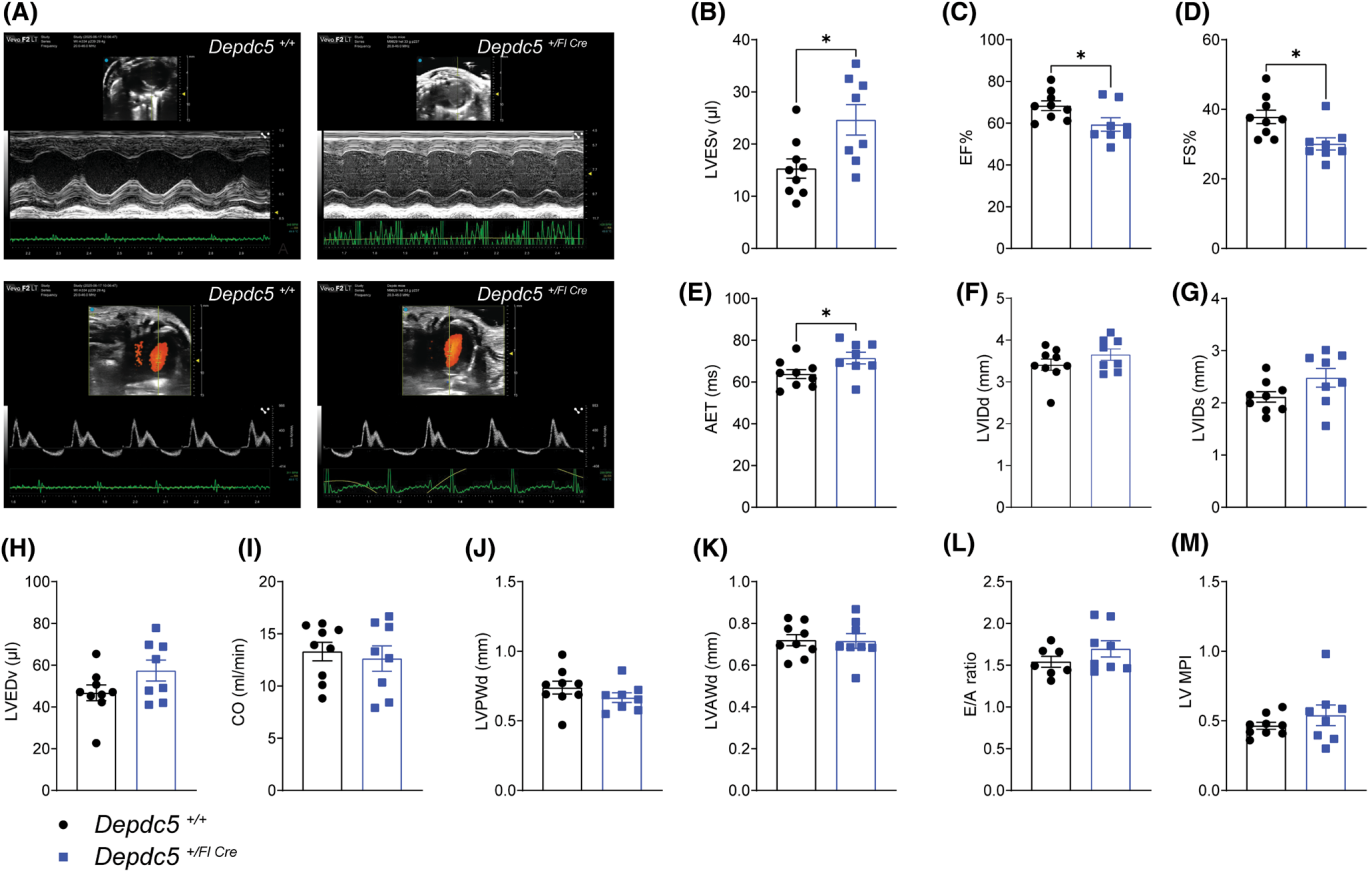
*Depdc5* ^*+/Fl Cre*^ mice exhibit early systolic dysfunction with preserved diastolic filling. (A) Representative M‐mode (top) and pulsed‐wave Doppler (bottom) echocardiographic images from 8‐month‐old *Depdc5* ^*+/+*^ (left) and *Depdc5* ^*+/Fl Cre*^ (right) mice. M‐mode images were obtained in the parasternal short‐axis view; Doppler images were acquired at the mitral inflow to assess diastolic filling (E and A waves). Quantified parameters include the following: (B) left ventricle (LV) end‐systolic volume (LVESv; *p* = .0146), (C) ejection fraction (EF%; *p* = .0354), (D) fractional shortening (FS%; *p* = .0105), (E) aortic ejection time (AET; *p* = .0439), (F) LV internal diameter in diastole (LVIDd; *p* = .2270), (G) LV internal diameter in systole (LVIDs; *p* = .0845), (H) LV end‐diastolic volume (LVEDv; *p* = .1038), (I) cardiac output (CO; *p* = .6583), (J) LV posterior wall thickness in diastole (LVPWd; *p* = .2432), (K) LV anterior wall thickness in diastole (LVAWd; *p* = .9328), (L) Mitral inflow E/A ratio (*p* = .2234), and (M) LV myocardial performance index (LVMPI; *p* = .3335). *Depdc5* ^*+/Fl Cre*^ mice exhibited significantly increased LVESv and prolonged AET, along with reduced EF% and FS%, indicative of impaired systolic performance. Other parameters, including ventricular dimensions, wall thickness, cardiac output, and mitral inflow velocities, were not significantly different between genotypes, suggesting preserved diastolic function. Data are presented as mean ± SEM. *n* = 8–9 mice per group. Statistical significance was determined using unpaired two‐tailed Student *t*‐test. *p < .05.

We assessed cardiac function using transthoracic echocardiography. M‐mode imaging and PW Doppler analysis revealed altered ventricular wall motion in *Depdc5* ^*+/Fl Cre*^ hearts compared to *Depdc5* ^*+/+*^ controls (Figure 2A). Quantification showed a significant increase in LVESv (*p* = .0146), indicating impaired systolic ejection (Figure 2B). EF% and FS% were reduced in *Depdc5* ^*+/Fl Cre*^ mice (*p* = .0354 and *p* = .0105, respectively; Figure 2C,D), indicating early contractile dysfunction. Aortic ejection time was prolonged in *Depdc5* ^*+/Fl Cre*^ hearts (*p* = .0439; Figure 2E), suggesting reduced ventricular efficiency. Although trends toward increased left ventricular internal dimension at end‐systole (LVIDs) and end‐diastole (LVIDd) were observed (Figure 2F,G), they did not reach significance (*p* = .2270 and *p* = .0845, respectively). Other parameters, including LVEDv (*p* = .1038), CO (*p* = .6583), and ventricular wall thickness (LV posterior wall thickness in diastole and LV anterior wall thickness in diastole; *p* = .2432 and *p* = .9328, respectively), were not different between genotypes, suggesting no evidence of cardiac hypertrophy (Figure 2H–K). Mitral inflow PW Doppler showed no difference in E/A ratio (*p* = .2234; Figure 2L), suggesting preserved diastolic filling. MPI, a composite measure of global cardiac function, was unchanged (*p* = .3335; Figure 2M). Together, these results indicate that *Depdc5* haploinsufficiency leads to systolic dysfunction and reduced ventricular contractility, whereas diastolic function and gross structural parameters remain largely intact.

### 3.4 *Depdc5* ^*+/Fl Cre*^ mice have increased levels of ventricular fibrosis

**FIGURE 3:**
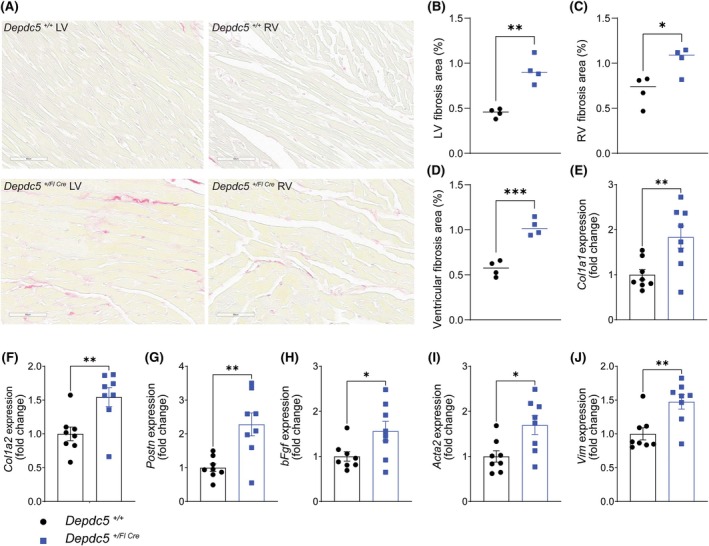
Increased cardiac fibrosis and fibrotic gene expression in *Depdc5* ^*+/Fl Cre*^ mice. (A) Representative images of Picrosirius Red‐stained heart sections from 8‐month‐old *Depdc5* ^*+/+*^ and *Depdc5* ^*+/Fl Cre*^ mice, showing collagen deposition (red) in the left ventricle (LV) and right ventricle (RV). Fibrotic regions are notably increased in *Depdc5* ^*+/Fl Cre*^ hearts. (B–D) Quantification of fibrotic area (%) in the LV (B), RV (C), and combined ventricles (D). *Depdc5* ^*+/Fl Cre*^ mice exhibit significantly increased fibrosis in all regions analyzed. (E–J) Quantitative reverse transcriptase polymerase chain reaction analysis of fibrosis‐related and mesenchymal gene expression in ventricular tissue. *Depdc5* ^*+/Fl Cre*^ mice show upregulation of *Col1a1* (E), *Col1a2* (F), *Postn* (G), *bFgf* (H), *Acta2* (I), and *Vim* (J), consistent with enhanced fibrotic remodeling. Data are presented as mean ± SEM. Sample sizes: *n* = 4 for histology, *n* = 8 for gene expression. *p < .05, **p < .01, ***p < .001. Statistical significance was determined by unpaired two‐tailed Student *t*‐test with Welch correction.

To assess whether *Depdc5* haploinsufficiency promotes cardiac fibrosis, we performed Picrosirius Red staining on ventricular tissue. *Depdc5* ^*+/Fl Cre*^ hearts exhibited increased interstitial collagen deposition in LV and RV (Figure 3A). Quantification showed increased fibrotic areas in the LV, RV, and total myocardium of *Depdc5* ^*+/Fl Cre*^ mice (Figure 3B–D). To gain deeper insight into fibrotic remodeling at the molecular level, we assessed the expression of extracellular matrix‐related genes and fibroblast activation genes in ventricular tissue by reverse transcriptase (RT)‐qPCR. We found increased mRNA abundance of *Col1a1* (collagen type I), *Col1a2* (collagen type 1 alpha 2), and *Postn* (periostin) in *Depdc5* ^*+/Fl Cre*^ hearts compared to control, suggesting enhanced collagen synthesis and active extracellular matrix remodeling (Figure 3E–G). Additionally, mRNA abundance of *bFgf* (basic fibroblast growth factor), *Acta2* (α‐smooth muscle actin), and *Vim* (vimentin) were elevated in *Depdc5* ^*+/Fl Cre*^ hearts compared to control (Figure 3H–J). Together, these findings suggest that *Depdc5* haploinsufficiency promotes fibrotic remodeling in mice at both histological and transcriptional levels.

### 3.5 *Depdc5^+/Fl Cre^* mouse ventricular cardiomyocytes exhibit increased *I*_Na_ density

**FIGURE 4:**
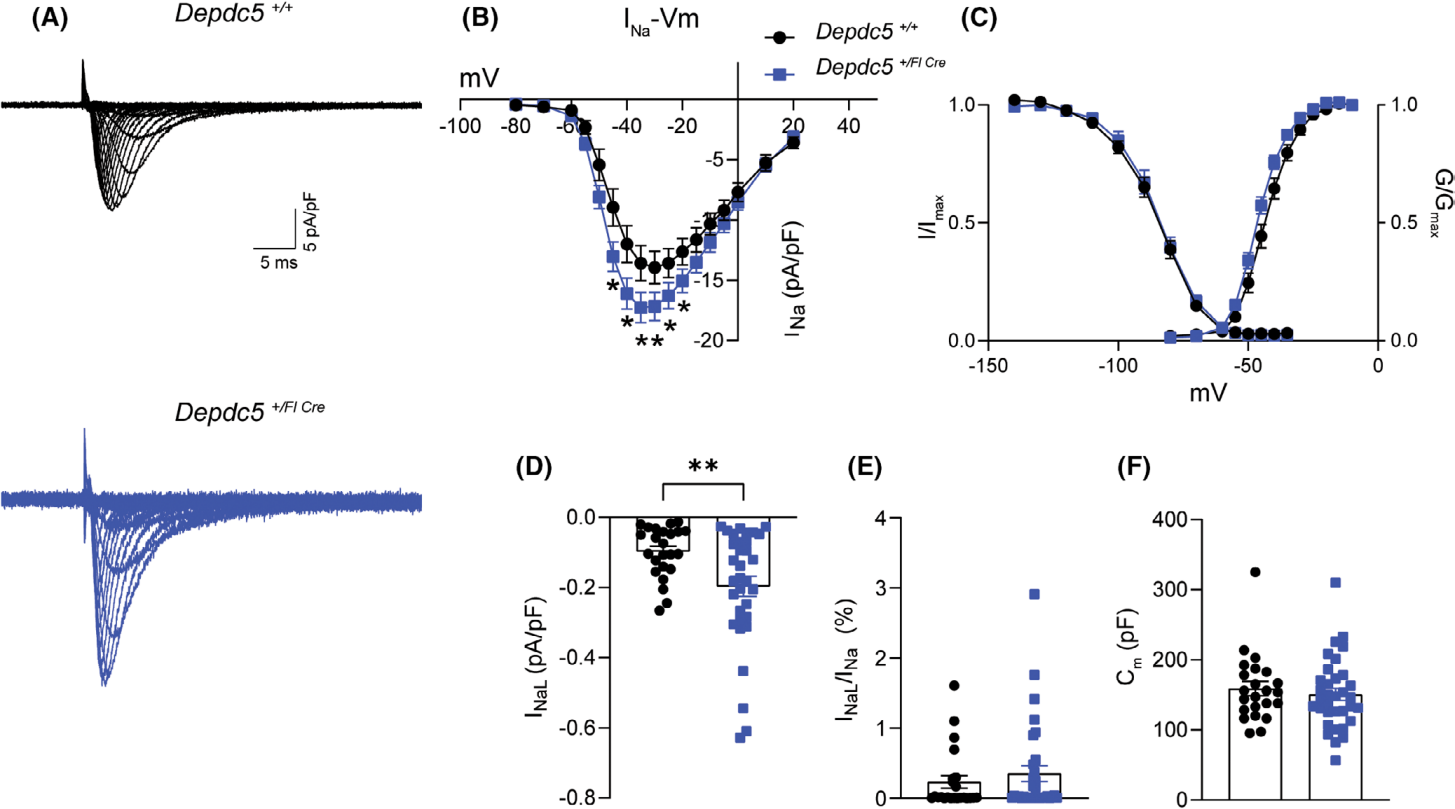
Sodium current (*I*_Na_) density is increased in acutely dissociated *Depdc5* ^*+/Fl Cre*^ cardiac myocytes (CMs). (A) Representative recordings of *I*_Na_ density in postnatal day 230–260 *Depdc5* ^*+/+*^ and *Depdc5* ^*+/Fl Cre*^ CMs. *Depdc5* ^*+/Fl Cre*^ CMs showed higher *I*_Na_ density compared to *Depdc5* ^*+/+*^. (B) Voltage–current (IV) relationship of *I*_Na_ density. Higher *I*_Na_ density was found at −40, −35, −30, −25, and − 20 mV in the *Depdc5* ^*+/Fl Cre*^ CMs compared to control. (C) Normalized activation and inactivation curves. No differences in voltage‐dependent properties were observed between genotypes. (D) Late sodium current density (*I*_NaL_) recorded at −20 mV. *I*_NaL_ density was increased in *Depdc5* ^*+/Fl Cre*^ CMs. (E) Normalized *I*_NaL_ density to *I*_Na_ density recorded at −20 mV from the IV curves. (F) Membrane capacitance (C_m_). Values represent mean ± SEM. *n* = 23 CMs, *n* = 4 mice for *Depdc5* ^*+/Fl Cre*^ mice and *n* = 37 CMs, *n* = 5 mice for *Depdc5* ^*+/Fl Cre*^ mice. *p < .05 compared to *Depdc5* ^*+/+*^, determined by one‐way analysis of variance followed by Tukey post hoc comparison test. Dots represent individual cells.

In addition to structural remodeling, electrical remodeling, or alterations in membrane ion channel function, can also contribute to cardiac arrhythmogenesis. To test this hypothesis, we performed patch‐clamp recordings in acutely isolated ventricular CMs. *Depdc5* ^*+/Fl Cre*^ CMs exhibited larger *I*_Na_ density compared to *Depdc5* ^*+/+*^ (Figure 4A). At the peak of the voltage–current curve, which occurred at −35 mV, *I*_Na_ was −17.3 ± 1.3 pA/pF in *Depdc5* ^*+/Fl Cre*^ CMs versus −13.6 ± 1.5 pA/pF in *Depdc5* ^*+/+*^ CMs (*p* < .05; Figure 4B). Increased *I*_Na_ density occurred without alterations in voltage‐dependent activation or inactivation properties (Figure 4C). Late sodium current (*I*_NaL_) density was increased in *Depdc5* ^*+/Fl Cre*^ CMs (−.20 ± .03 pA/pF) compared to controls (−.09 ± .01 pA/pF, *p* < .05; Figure 4D), although the ratio of *I*_NaL_:*I*_Na_ was not different between genotypes (Figure 4E). Membrane capacitance was similar between genotypes, consistent with the observed unaltered HW/BW ratio (Figure 4F).

### 3.6 *Depdc5^+/Fl Cre^* mouse ventricular cardiomyocytes exhibit increased *I*_to_ density

**FIGURE 5:**
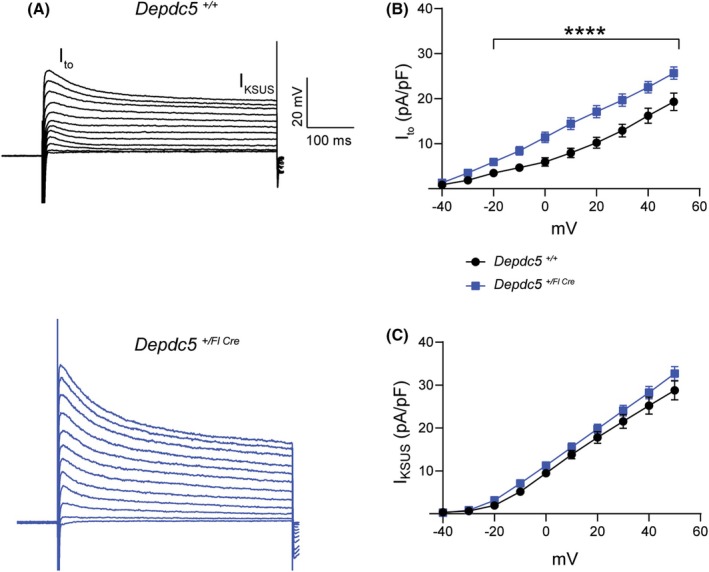
Transient outward potassium current (*I*_to_) density is upregulated in *Depdc5* ^*+/Fl Cre*^ cardiac myocytes (CMs). (A) Representative potassium current (*I*_K_) recordings from postnatal day 230–260 *Depdc5* ^*+/+*^ and *Depdc5* ^*+/Fl Cre*^ CMs. *Depdc5* ^*+/Fl Cre*^ CMs showed increased *I*_to_ density. (B) Voltage–current (IV) relationship for *I*_to_ density. Increased *I*_to_ density occurred at membrane potentials of −20, 0, 10, 20, 30, 40, and 50 mV. (C) IV relationship for sustained potassium current (*I*_KSUS_) density. Values represent mean ± SEM. *n* = 16 CMs, *n* = 5 mice for *Depdc5* ^*+/+*^ and *n* = 21 CMs, *n* = 5 mice for *Depdc5* ^*+/Fl Cre*^. ****p < .0001 against *Depdc5* ^*+/+*^, determined by one‐way analysis of variance followed by Tukey post hoc comparison test.

*Depdc5* ^*+/Fl Cre*^ CMs showed a larger *I*_to_ density compared to *Depdc5* ^*+/+*^ (Figure 5A). *I*_to_ density was increased in *Depdc5* ^*+/Fl Cre*^ CMs across membrane potentials from −20 to 50 mV (Figure 5B). *Depdc5* ^*+/Fl Cre*^ CMs also showed reduced inward rectifier potassium current (*I*_K1_) density compared to controls at hyperpolarizing currents of −120 and − 110 mV (Figure S1). However, no differences in *I*_K1_ density were observed within the RMP range (−85 to −75 mV), and there were no differences in *I*_KSUS_ (Figure 5C). *I*_Ca,L_, which slows membrane repolarization by opposing outward potassium currents, was not different between genotypes (Figure S2).

### 3.7 *Depdc5* ^*+/Fl Cre*^ CMs exhibit shortened AP duration

**FIGURE 6:**
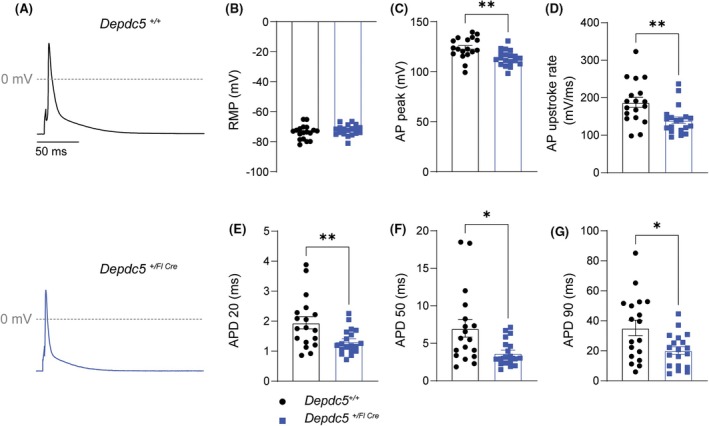
*Depdc5* ^*+/Fl Cre*^ cardiac myocyte (CM) action potential (AP) waveforms show slowed membrane depolarization and rapid repolarization. (A) Representative AP recordings from in postnatal day 230–260 *Depdc5* ^*+/+*^ and *Depdc5* ^*+/Fl Cre*^ CMs. APs were recorded at 1.0 Hz. (B) Resting membrane potential (RMP). (C) AP peak. (D) AP upstroke. Both AP peak and AP upstroke were reduced in *Depdc5* ^*+/Fl Cre*^ CMs. (E) Action potential duration (APD) at 20% of membrane repolarization (APD 20). (F) APD at 5% of membrane repolarization (APD 50) (G) APD at 90% of membrane repolarization (APD 90). *Depdc5* ^*+/Fl Cre*^ CMs showed reduced APD at all percentages. Values represent mean ± SEM. *n* = 18 CMs, *n* = 4 mice for *Depdc5* ^*+/+*^ and *n* = 19 CMs, *n* = 4 mice for *Depdc5* ^*+/Fl Cre*^. *p < .05, **p < .01 compared to *Depdc5* ^*+/+*^, determined by one‐way analysis of variance followed by Tukey post hoc comparison test. Dots represent individual cells.

Figure 6A shows representative AP recordings in acutely isolated ventricular CMs. We found no genotypic differences in RMP (Figure 6B). However, *Depdc5* ^*+/Fl Cre*^ CMs showed reduced AP peak amplitude and reduced AP upstroke velocity compared to *Depdc5* ^*+/+*^ (Figure 6C,D). *Depdc5* ^*+/Fl Cre*^ CMs showed shorter action potential duration (APD), or accelerated membrane repolarization, throughout the AP waveform (Figure 6E–G), consistent with the observed upregulation of *I*_to_ density (Figure 5) in these cells. In contrast, the reductions in AP amplitude and upstroke in *Depdc5* ^*+/Fl Cre*^ CMs were not predicted from the observed increase in *I*_Na_ density (Figure 4). This discrepancy suggests that impaired membrane depolarization may occur despite elevated *I*_Na_ density, potentially due to the dominant influence of enhanced *I*_to_. Previous work using dynamic clamp in human embryonic kidney cells suggested a similar mechanism,^41^ although further studies are needed for confirmation in mouse CMs.

### 3.8 Gene expression profiling reveals ion channel remodeling in *Depdc5* ^*+/Fl Cre*^ hearts

To identify potential molecular mechanisms of altered ionic currents, we performed RT‐qPCR on isolated ventricular tissues. *Depdc5* expression was reduced in *Depdc5* ^*+/Fl Cre*^ mouse ventricles, confirming effective heterozygous deletion (Figure S3A). mRNA abundances of the VGSC genes, *Scn1b, Scn2b*, and *Scn5a*, were not different between genotypes (Figure S3B–D), suggesting alternative, possibly posttranslational, mechanisms for increased *I*_Na_ density. In contrast, mRNA abundance of *Kcnd2*, encoding K_v_4.2, was increased in *Depdc5* ^*+/Fl Cre*^ ventricles, in agreement with elevated *I*_to_ density observed in patch‐clamp recordings (Figure S3E, Figure 5). mRNA abundances of genes coding Kir2.1 (*Kcnj2*) and Ca_v_1.2 (*Cacna1c*), which underlie *I*_K1_ and *I*_Ca,L_, respectively, were not different between genotypes (Figure S3F,G). These findings suggest that, whereas *I*_Na_ density increases are likely mediated through posttranscriptional mechanisms, the increase in *I*_to_ density is driven by transcriptional changes secondary to *Depdc5* haploinsufficiency.

### 3.9 *Depdc5* ^*+/Fl Cre*^ mouse ventricles have increased levels of Nav1.5 and Kv4.2 channel proteins

We performed Western blot analyses to determine whether the observed increases in *I*_Na_ and *I*_to_ in *Depdc5* ^*+/Fl Cre*^ ventricles correlated with increased levels of channel protein. Figure S4 shows representative Western blots of anti‐Nav1.5 and anti‐Kv4.2 protein expression in ventricular lysates from *Depdc5* ^*+/+*^ and *Depdc5* ^*+/Fl Cre*^ mice. Anti‐GAPDH was used for normalization of fluorescence signal densitometry. Quantification of protein band intensities demonstrated significantly increased Nav1.5 and Kv4.2 protein abundance in *Depdc5* ^*+/Fl Cre*^ ventricles. Although increases in *I*_Na_ in *Depdc5* ^*+/Fl Cre*^ ventricles do not correspond to changes in *Scn5a* mRNA abundance, increased Nav1.5 protein expression may be the result of changes in mRNA or protein stability. Taken together with RT‐qPCR data showing increased mRNA abundance of *Kcnd2*, electrophysiological recordings showing increased *I*_Na_ and *I*_to_, and shortened APD, these data confirm that *Depdc5* haploinsufficiency increases functional expression of depolarizing and repolarizing cardiac ion channels.

## 4 DISCUSSION

Our previous body of work revealed cardiac phenotypes in mouse and human models of channelopathy‐linked DEEs.^14^, ^15^, ^17^, ^18^, ^19^, ^40^ Here, we demonstrate that systemic haploinsufficiency of the non‐ion‐channel gene, *Depdc5*, also results in altered CM excitability, including increased sodium and potassium channel proteins and function, ventricular arrhythmia, systolic dysfunction, and ventricular fibrosis. Importantly, these effects occur in the absence of seizures and SUDEP in this mouse model, implying cell autonomous effects of *Depdc5* haploinsufficiency in the heart. Thus, non‐ion‐channel‐linked DEE gene variants may also increase susceptibility to cardiac disease and SCD.

*DEPDC5* is part of the mTOR signaling pathway, which is critical in the heart as well as the brain, where it plays essential roles in cardiac development and the cardiac adaptation to stress and thus has been identified as a promising therapeutic target for heart disease.^34^, ^42^, ^43^ mTOR pathway dysfunction can lead to maladaptive cardiac hypertrophy and remodeling, including dilated cardiomyopathy and myocardial fibrosis. Although *Mtor* deletion is clearly more severe than *Depdc5* deletion in mice, and the downstream role of *Mtor* in cardiac arrhythmias is not well understood, previous data derived from cardiac‐specific *Mtor* null mice may be informative here. Cardiac‐specific *Mtor* deletion in mice resulted in almost complete embryonic lethality,^38^ and early postnatal deletion caused dilated cardiomyopathy.^44^ Inducible *Mtor* deletion in adult mice resulted in impaired hypertrophic responses and accelerated heart failure progression when animals were subjected to pressure overload.^45^ Modulation of cardiac ion channel activity has been implicated in the mTOR pathway. For example, overactivation of mTORC1 activity secondary to lipotoxicity has been suggested to lead to increased rapid delayed rectifier current and shortened APDs in atrial CMs secondary to altered hERG1a/b expression.^46^ Although more detailed work is needed to test the hypothesis, mTORC1 signaling has been postulated to function as a “voltage‐sensor” via modulation of multiple ion channel mRNAs, including those encoding the ion channel proteins Kv1.1, Kv1.2, and Kvβ2, as well as Cav1.2, Cav1.3, and α2δ2, to modulate synaptic function in brain.^47^ mTOR hyperactivation in obesity is proposed to induce increased K_ATP_ channel activity in hypothalamic neurons.^48^ Cortical pyramidal neurons recorded from *Depdc5* ^*+/−*^ rat brain slices have hyperpolarized RMPs and decreased firing rates compared to wild type, changes proposed to occur via potentiation of potassium currents.^49^ Thus, it is reasonable to predict that similar changes in ion channel function occur in the heart, in agreement with the present work.

We have demonstrated intrinsic deficits in CM excitability and cardiac arrhythmias in three transgenic mouse models of channelopathy‐linked DEE. Acutely isolated *Scn1a* ^*+/R1407X*^ DS mouse ventricular CMs showed increased *I*_Na_ and *I*_Na,L_, AP prolongation, and increased incidence of delayed afterdepolarizations (DADs).^17^ ECGs showed prolonged QT intervals.^17^ Ventricular CMs from an *Scn8a* gain‐of‐function mouse showed AP prolongation, prolonged calcium transients, and aberrant calcium release that were tetrodotoxin‐sensitive and SN‐6‐sensitive, implicating Na_v_1.6‐generated *I*_Na_ and subsequent activation of reverse Na/Ca exchange in arrhythmogenesis.^15^ Arrhythmias could be induced with epinephrine plus caffeine to mimic an ictal sympathetic surge. Consistent with our work, a patient with an *SCN8A*‐linked DEE variant was reported with cardiac arrhythmias and ictal asystole.^50^ *SCN1B* variants are linked to human cardiac disease in addition to DEE.^51^ *Scn1b* ^*−/−*^ mouse ventricular CMs have increased *I*_Na_ and *I*_Na,L_, increased *I*_to_, decreased *I*_Ca,L_, AP prolongation, prolonged calcium transients, and increased incidence of DADs.^18^, ^52^, ^53^ *Scn5a*/Nav1.5 and *Scn3a* expression, as well as ^3^H‐saxitoxin binding, which measures levels of tetrodotoxin‐sensitive VSGC expression, are increased in the *Scn1b* ^−/−^ heart. Like *Scn8a* mice, AP prolongation and aberrant calcium release in *Scn1b* ^−/−^ mice are tetrodotoxin‐sensitive. *Scn1b* ^*−/−*^ mice also have pacing‐induced atrial fibrillation.^19^

In neuron‐specific *Depdc5* ^*−/−*^ mice, spontaneous seizures resulting in SUDEP were not preceded by detectable cardiac arrhythmias.^54^ In a separate study, *Depdc5* deletion in a subpopulation of mouse forebrain excitatory neurons resulted in frequent generalized tonic–clonic seizures in young adult mice, which eventually developed SUDEP.^37^ Interictal respiratory dysregulation and ictal apneas were recorded prior to terminal cardiac asystole.^37^ Importantly, in both of these studies, *Depdc5* expression in the heart was intact in the experimental animals, whereas *DEPDC5* patients express mutant proteins in all tissues. Mice with simultaneous deletion of *Depdc5* and *Tsc1* in both cardiac and skeletal muscle showed impaired cardiac performance, including reduced ejection fraction and fractional shortening, similar to our results, although the two models are difficult to compare directly.^55^ Evaluation of 14 epilepsy patients with pathogenic *DEPDC5* variants revealed no evidence for baseline ECG, echocardiographic, or stress testing parameters that would predict sudden death, and postmortem examination of one *DEPDC5* SUDEP case failed to reveal structural cardiac abnormalities.^54^ However, in some VGSC disorders such as Brugada syndrome, the diagnostic ECG pattern is variably present over time, and the diagnosis often requires provocative testing using VGSC‐blocking pharmacology.

Whole exome sequencing of a proband with Brugada syndrome and aborted SCD and three of his family members with ECGs (spontaneous or procainamide‐induced) consistent with Brugada syndrome but no cardiac structural abnormalities showed a rare, conserved autosomal dominant variant in *DEPDC5*, c.G347T‐p.R116L.^56^ Of note, another family member carrying this variant developed epilepsy. Assuming that this variant causes loss of *DEPDC5* function in cardiac myocytes, an increase in *I*_to_ similar to our observations in *Depdc5* ^*+/Fl Cre*^ mouse CMs could contribute to the observed Brugada phenotype. Although differences in cardiac electrophysiology between humans and mice complicate the comparison, these findings strengthen the potential link between *DEPDC5*, epilepsy, and cardiac excitability in humans, although additional work is needed before wide‐scale clinical cardiac investigation is recommended to patients with *DEPDC5* variants.

Importantly, the findings presented here expand our previous work showing cardiac arrhythmia in multiple mouse and human channelopathy DEE models to now include a mouse model of a non‐ion‐channel DEE variant. In mice, *Depdc5* variants impact the heart as well as the brain, strengthening the hypothesis that cardiac arrhythmias may contribute to the complex presentation of DEE. Although this work may have implications for *DEPDC5* patients, critical differences between mouse and human cardiac physiology complicate the translation of mouse data to human disease.^57^ Future investigations of *Depdc5* function in higher vertebrate models that more accurately mimic human physiology, as well as longitudinal patient natural history studies that monitor cardiovascular health, will be required to test the hypothesis that DEE variants resulting in *DEPDC5* haploinsufficiency may predispose patients to cardiac arrhythmias.

## AUTHOR CONTRIBUTIONS

Alexander M. Greiner and Barry London provided the mouse model, which was then bred and maintained by Chunling Chen. Roberto Ramos‐Mondragon conducted patch‐clamp experiments to record voltage‐dependent sodium, calcium, and potassium currents, as well as APs in isolated mouse CMs. Roberto Ramos‐Mondragon also performed programmed electrical stimulation in anesthetized mice and contributed to the experimental design. Shuyun Wang performed cardiac myocyte isolation, Picrosirius Red staining, imaging, qPCR, Western blots, and echocardiography and data analysis. Qinghua Liu assisted with the recording of potassium currents and APs in isolated CMs. Abigail M. Marx and Maya Shih processed and analyzed patch‐clamp data and conducted statistical analyses in a blinded fashion. Roberto Ramos‐Mondragon, Shuyun Wang, Barry London, Jack M. Parent, and Lori L. Isom cowrote the manuscript.

## CONFLICT OF INTEREST STATEMENT

The authors declare no conflicts of interest. We confirm that we have read the Journal's position on issues involved in ethical publication and affirm that this report is consistent with those guidelines.

## Supporting information


**Figure S1.** Inward rectifier potassium current (*I*_K1_) density recordings. (A) Voltage‐clamp protocol used to record *I*_K1_. Inward currents were measured before and after perfusion with barium (Ba^2+^). The Ba^2+^‐sensitive *I*_K1_ component was obtained by subtracting trace *b* from trace *a* (*a* − *b*). (B) Current–voltage relationship of *I*_K1_ density, illustrating the Ba^2+^‐sensitive component. *Depdc5* ^*+/Fl Cre*^ cardiac myocytes (CMs) exhibited significantly reduced *I*_K1_ density at hyperpolarized membrane potentials of −120 and − 110 mV compared to *Depdc5* ^*+/+*^ controls. No significant differences in *I*_K1_ density were observed within the range of resting membrane potential (−85 to −75 mV). *I*_K1_: *n* = 24 CMs, *n* = 5 mice for *Depdc5* ^*+/+*^ and *n* = 20 CMs, *n* = 3 mice for *Depdc5* ^*+/Fl Cre*^.
**Figure S2.** I_CaL_ density is unaltered in *Depdc5* ^*+/Fl Cre*^ cardiac myocytes (CMs). (A) Representative recordings of L‐type calcium current (I_CaL_) density from *Depdc5* ^*+/+*^ and *Depdc5* ^*+/Fl Cre*^ CMs. (B) *I*_CaL_ density recorded from single pulse to 10 mV that was applied before the application of current–voltage protocol. (C) Voltage–current relationship of *I*_CaL_ density. No significant differences in *I*_CaL_ density were observed between genotypes. Values represent mean ± SEM. *n* = 16 CMs, *n* = 3 mice from *Depdc5* ^*+/Fl Cre*^ and *n* = 15 CMs, *n* = 3 mice from *Depdc5* ^*+/Fl Cre*^.
**Figure S3.** Altered ion channel gene expression in *Depdc5* ^*+/Fl Cre*^ hearts. (A) *Depdec5* mRNA abundance is reduced by approximately 50% in *Depdc5* ^*+/Fl Cre*^ hearts. (B) mRNA abundance of *Scn5a*. (C) mRNA abundance of *Scn1b*. (D) mRNA abundance of *Scn2b*. (E) mRNA abundance of *Kcnd2*. *Depdc5* ^*+/Fl Cre*^ hearts showed increased mRNA abundance of *Kcnd2*. (F) mRNA abundance of *Kcnj2*. (G) mRNA abundance of *Cacna1c*. Values represent mean ± SEM; *n* = 8 per group. ***p < .001, ****p < .0001. Statistical significance was determined by unpaired two‐tailed Student *t*‐test.
**Figure S4.** *Depdc5* ^*+/Fl Cre*^ mouse ventricles show increased levels of Nav1.5 and Kv4.2 channel proteins. (A, C) Representative Western blots showing Nav1.5 and Kv4.2 protein expression in ventricular lysates from *Depdc5* ^*+/+*^ and *Depdc5* ^*+/Fl Cre*^ mice. Glyceraldehyde‐3‐phosphate dehydrogenase (GAPDH) was used for normalization. (B, D) Quantification of protein band intensities demonstrates significantly increased Nav1.5 and Kv4.2 protein abundance in *Depdc5* ^*+/Fl Cre*^ ventricles. Data are presented as mean ± SEM; *n* = 4 per group. Statistical significance was determined by unpaired two‐tailed Student *t*‐test. **p < .01, ***p < .001.
**Table S1.** Heart weight to body weight relationship and electrocardiographic parameters in anesthetized mice. QT was corrected using Mitchell's formula (QTc = QT/√RR/100). Data are presented as mean ± SEM.
**Table S2.** Voltage‐dependent activation and inactivation for *I*_Na_. Data are presented as mean ± SEM. *p < .05 against using one‐way analysis of variance with Tukey post hoc comparison test.
**Table S3.** Programmed electrical stimulation parameters. Data are presented as mean ± SEM. *p < .05 using one‐way analysis of variance with Tukey post hoc comparison test.

## Data Availability

Data and animals are available on request from the authors.
