# Supplementary material for: Cardiac remodeling and arrhythmia in a mouse model of Depdc5 haploinsufficiency
Source: Epilepsia. 2026 Apr 9;67(7):3738–52. doi: 10.1002/epi.70244 (PMC13360907; doi:10.1002/epi.70244)
Supplement: Supplementary file 1 — Figure S1. Inward rectifier potassium current (I K1) density recordings. (A) Voltage‐clamp protocol used to record I K1. Inward currents were measured before and after perfusion with barium (Ba2+). The Ba2+‐sensitive I K1 component was obtained by subtracting trace b from trace a (a − b). (B) Current–voltage relationship of I K1 density, illustrating the Ba2+‐sensitive component. Depdc5 +/Fl Cre cardiac myocytes (CMs) exhibited significantly reduced I K1 density at hyperpolarized membrane potentials of −120 and − 110 mV compared to Depdc5 +/+ controls. No significant differences in I K1 density were observed within the range of resting membrane potential (−85 to −75 mV). I K1: n = 24 CMs, n = 5 mice for Depdc5 +/+ and n = 20 CMs, n = 3 mice for Depdc5 +/Fl Cre . Figure S2. ICaL density is unaltered in Depdc5 +/Fl Cre cardiac myocytes (CMs). (A) Representative recordings of L‐type calcium current (ICaL) density from Depdc5 +/+ and Depdc5 +/Fl Cre CMs. (B) I CaL density recorded from single pulse to 10 mV that was applied before the application of current–voltage protocol. (C) Voltage–current relationship of I CaL density. No significant differences in I CaL density were observed between genotypes. Values represent mean ± SEM. n = 16 CMs, n = 3 mice from Depdc5 +/Fl Cre and n = 15 CMs, n = 3 mice from Depdc5 +/Fl Cre . Figure S3. Altered ion channel gene expression in Depdc5 +/Fl Cre hearts. (A) Depdec5 mRNA abundance is reduced by approximately 50% in Depdc5 +/Fl Cre hearts. (B) mRNA abundance of Scn5a. (C) mRNA abundance of Scn1b. (D) mRNA abundance of Scn2b. (E) mRNA abundance of Kcnd2. Depdc5 +/Fl Cre hearts showed increased mRNA abundance of Kcnd2. (F) mRNA abundance of Kcnj2. (G) mRNA abundance of Cacna1c. Values represent mean ± SEM; n = 8 per group. ***p < .001, ****p < .0001. Statistical significance was determined by unpaired two‐tailed Student t‐test. Figure S4. Depdc5 +/Fl Cre mouse ventricles show increased levels of Nav1.5 and Kv4.2 channel proteins. (A, [file EPI-67-3738-s001.docx]

**Supplemental Materials**

**Cardiac Remodeling and Arrhythmia in a Mouse Model of *Depdc5* Haploinsufficiency**

Roberto Ramos-Mondragon^1^, Shuyun Wang^1^, Qinghua Liu^1^, Chunling Chen^1^, Alexander M. Greiner^2^, Abigail M. Marx^1^, Maya Shih^1^, Jack M. Parent^3,4,5^, Barry London^2^, and Lori L. Isom^1,3^

Departments of Pharmacology^1^ or Neurology^3^, University of Michigan Medical School, Ann Arbor, MI 48109

^2^Department of Internal Medicine – Division of Cardiovascular Medicine, University of Iowa Carver College of Medicine, Iowa City, IA 52242

^4^Michigan Neuroscience Institute, University of Michigan Medical School, Ann Arbor, MI 48109

^5^VA Ann Arbor Healthcare System, Ann Arbor, MI 48105

Correspondence to: Lori L. Isom, Ph.D., Department of Pharmacology, University of Michigan Medical School, Ann Arbor, MI 48109, [lisom@umich.edu](mailto:lisom@umich.edu), 734-936-3050

**Supplemental Figures and Figure Legends:**


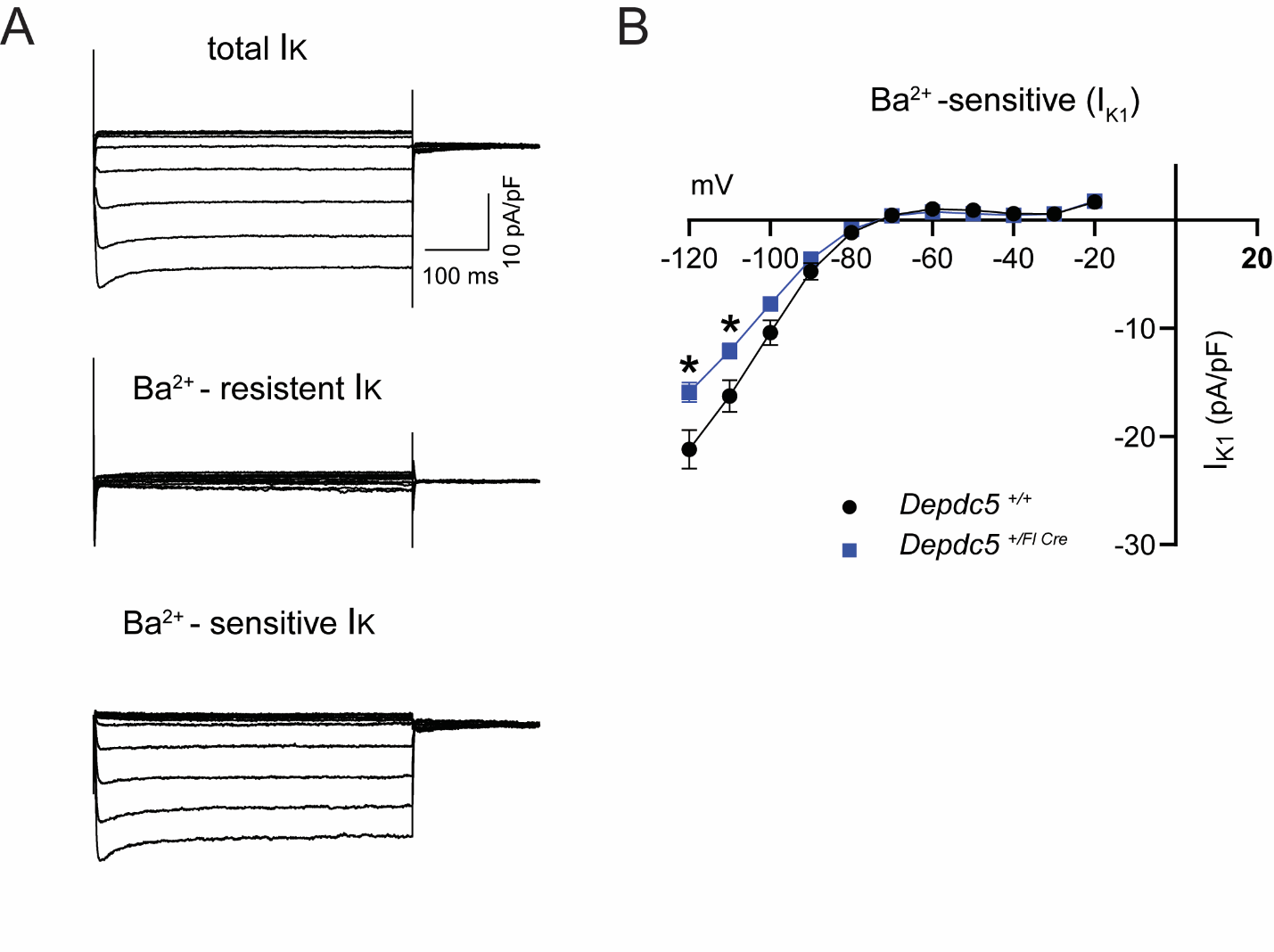


**Figure S1. Inward rectifier potassium currents (I_K1_) density recordings.** (A) Voltage-clamp protocol used to record I_K1_. Inward currents were measured before and after perfusion with barium (Ba²⁺). The Ba²⁺-sensitive I_K1_ component was obtained by subtracting trace b from trace a (a – b). (B) Current–voltage (I–V) relationship of I_K1_ density, illustrating the Ba²⁺-sensitive component. *Depdc5^+/Fl Cre^* CMs exhibited significantly reduced I_K1_ density at hyperpolarized membrane potentials of -120 and -110 mV compared to *Depdc5^+/+^* controls. No significant differences in I_K1_ density were observed within the range of resting membrane potential (-85 to -75 mV). I_K1_: n= 24 CMs, N=5 mice for *Depdc5^+/+^* and n= 20 CMs, N=3 mice for *Depdc5^+/Fl Cre^*.


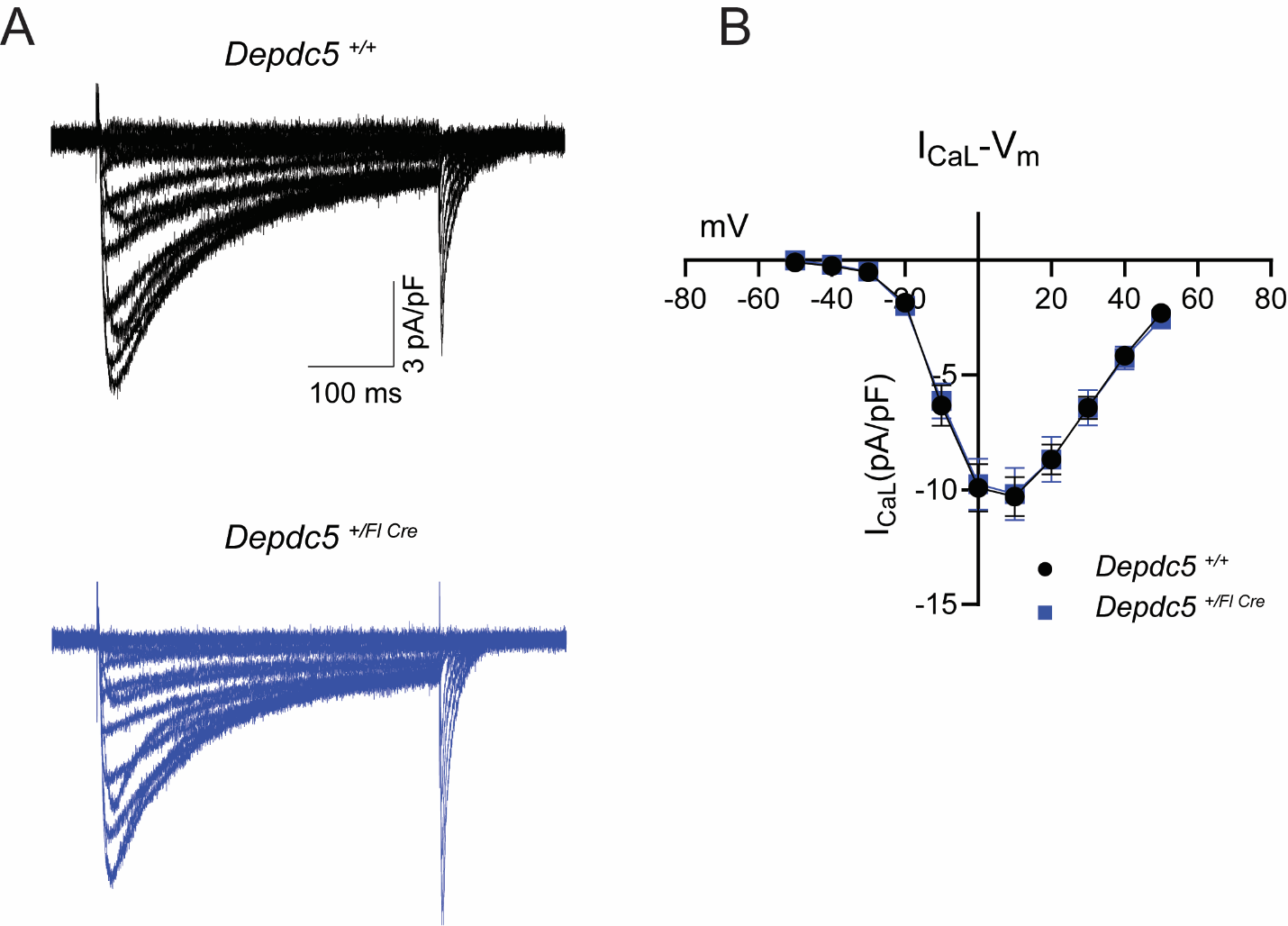


**Figure S2.** **I_CaL_ density is unaltered in *Depdc5^+/Fl Cre^* CMs.** (A) Representative recordings of L-type calcium current (I_CaL_) density from *Depdc5^+/+^* and *Depdc5^+/Fl Cre^* CMs. (B) I_CaL_ density recorded from single pulse to 10 mV that was applied before the application of current-voltage protocol. (C) Voltage-current relationship of I_CaL_ density. No significant differences in I_CaL_ density were observed between genotypes. Values represent mean ± SEM. n= 16 CMs, N=3 mice from *Depdc5^+/Fl Cre^* and n= 15 CMs, N=3 mice from *Depdc5^+/Fl Cre^*.


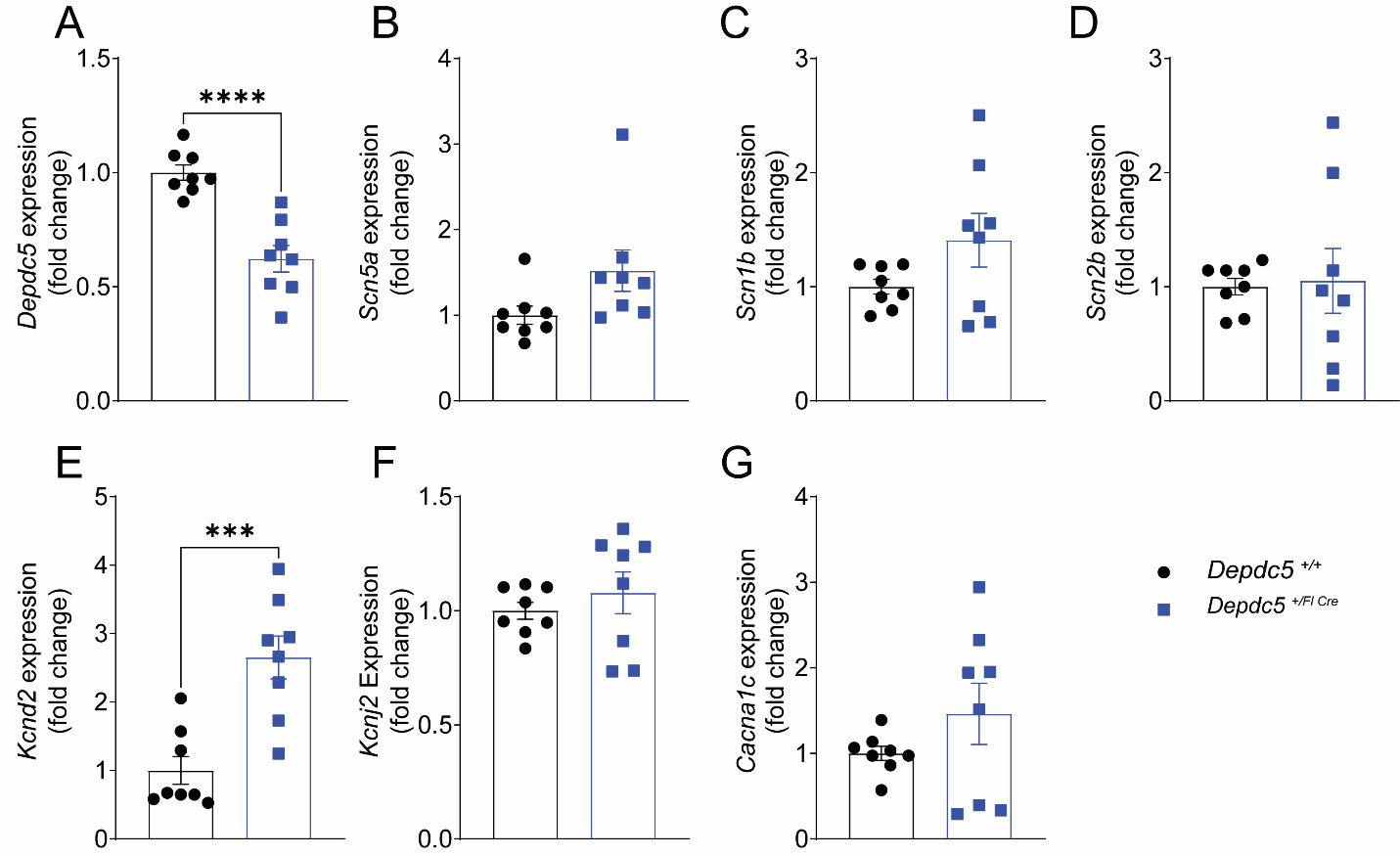


**Figure S3.** **Altered ion channel gene expression in *Depdc5^+/Fl Cre^* hearts.** (A) *Depdec5* mRNA abundance is reduced by approximately 50% in *Depdc5^+/Fl Cre^* hearts. (B) mRNA abundance of *Scn5a*. (C) mRNA abundance of *Scn1b*. (D) mRNA abundance of *Scn2b*. (E) mRNA abundance of *Kcnd2*. *Depdc5^+/Fl Cre^* hearts showed increased mRNA abundance of *Kcnd2*. (F) mRNA abundance of *Kcnj2*. (G) mRNA abundance of *Cacna1c*. * Values represent mean ± SEM; n = 8 per group. ****p < 0.001*, *****p < 0.0001*. Statistical significance was determined by unpaired two-tailed Student’s t-test.

**
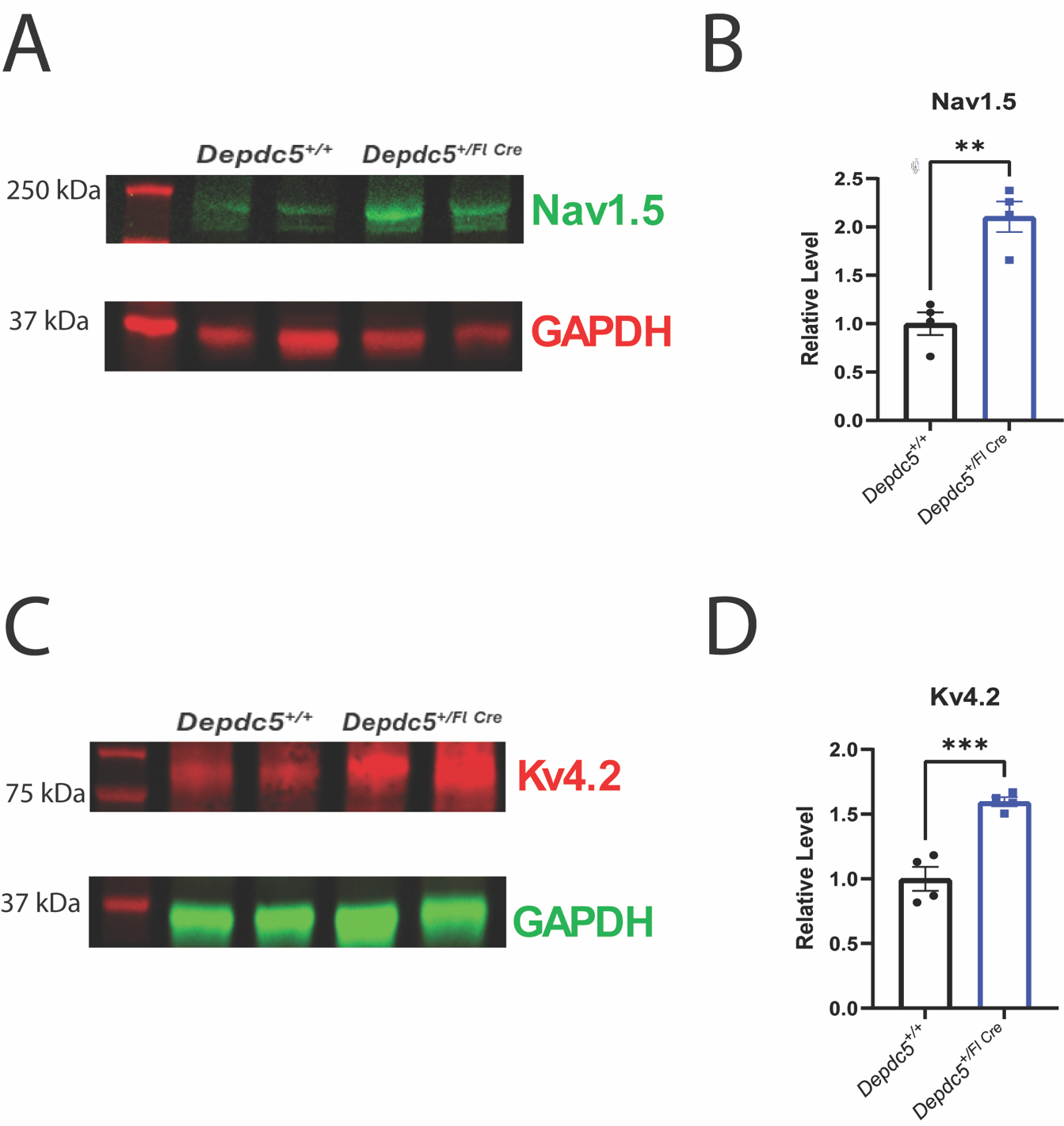
**

**Figure S4. *Depdc5^+/Fl Cre^* mouse ventricles show increased levels of Nav1.5 and Kv4.2 channel proteins.** (A, C) Representative Western blots showing Nav1.5 and Kv4.2 protein expression in ventricular lysates from *Depdc5^+/+^* and *Depdc5^+/Fl Cre^* mice. GAPDH was used for normalization. (B, D) Quantification of protein band intensities demonstrates significantly increased Nav1.5 and Kv4.2 protein abundance in *Depdc5^+/Fl Cre^* ventricles. *Data presented as mean ± SEM; n = 4 per group. Statistical significance was determined by unpaired two-tailed Student’s t-test. ***p < 0.01*, ****p < 0.001*.

**Supplemental Tables:**

|  | *Depdc5^+/+^* | *Depdc5^+/Fl Cre^* |
| --- | --- | --- |
| Body weight (g): | 25.9 ± 3.6 | 26.1 ± 1.7 |
| Heart weight (mg): | 173.4 ± 32.9 | 176.6 ± 22.1 |
| HW/BW (mg/g): | 6.6 ± 0.5 | 6.8 ± 0.7 |
| ECG-I | | |
| HR (bpm) | 495.5±42.7 | 483.7±36.9 |
| P wave (ms) | 9.2±1.1 | 10.5±1.7 |
| PR-I (ms) | 40.6±5.6 | 38.4±9.2 |
| QRS-I (ms) | 9.2±1,5 | 8.3±2.2 |
| QT-I (ms) | 38.5±7.1 | 37.3±9.2 |
| QTc-I (ms) | 34.7±5.3 | 33.3±8.1 |
| ECG-II | | |
| HR (bpm) | 507.3±49.3 | 485.7±36.1 |
| P wave (ms) | 9.5±2.1 | 11.9±2.2 |
| PR-I (ms) | 42.6±2.1 | 43.5±2.7 |
| QRS-I (ms) | 10.0±1.2 | 10.5±1.2 |
| QT-I (ms) | 44.3±6.6 | 47.8±4.6 |
| QTc-I (ms) | 40.4±5.5 | 42.8±3.3 |
| N = | 18 | 17 |

**Table S1.** **Heart weight to body weight relationship and ECG parameters in anesthetized mice.** QT was corrected using Mitchell’s formula (QTc= QT/ √RR/100). Data are presented as mean ± SEM.

|  | *Depdc5^+/+^* | *Depdc5^+/Fl Cre^* | *p-value* |
| --- | --- | --- | --- |
| Voltage-dependent activation |  |  |  |
| G_max_ (mV) | 35.7 ± 3.0 | 45.8 ± 2.6* | *p<0.05* |
| *V*_1/2_ (mV) | -42.9 ± 1.1 | -46.0 ± 0.7 |  |
| V_rev_ (mV) | 37.1 ± 4.0 | 29.5 ± 2.0 |  |
| Voltage-dependent inactivation |  |  |  |
| I_max_ (pA) | 1753.0 ± 131.8 | 2182.0 ± 185.7 |  |
| *V*_1/2_ (mV) | -86.02 ± 1.5 | -86.02 ± 1.6 |  |
| Cm (1) | 159.0 ± 10.07 | 150.8 ± 8.6 |  |
| n= | 23 | 37 |  |

**Table S2.** **Voltage dependent activation and inactivation for I_Na_.** Data are presented as mean ± SEM*. *p<0.05* against using One-Way Anova with Tukey’s post-hoc comparison test.

|  | *Depdc5^+/+^* | *Depdc5^+/Fl Cre^* |
| --- | --- | --- |
| SNRT_100_ (ms) | 159.3 ± 9.8 | 165.3 ± 9.2 |
| SNRT_80_ (ms) | 170.7 ± 8.4 | 189.8 ± 10.1 |
| VERP_100_ (ms) | 20.6 ± 1.0 | 21.4 ± 1.7 |
| VERP_80_ (ms) | 23.2 ± 1.3 | 21.8 ± 1.4 |
| VT duration (s) | 0.32 ± 0.02 | 0.44 ± 0.001* |
| N= | 13 | 13 |

**Table S3.** **Programmed electrical stimulation parameters.** Data are presented as mean ± SEM. **p<0.05* using a One-Way Anova with Tukey’s post-hoc comparison test.
